# DCMC as a Promising Alternative to Bentonite in White Wine Stabilization. Impact on Protein Stability and Wine Aromatic Fraction

**DOI:** 10.3390/molecules26206188

**Published:** 2021-10-14

**Authors:** Francesco Saracino, João Brinco, Diana Gago, Marco Gomes da Silva, Ricardo Boavida Ferreira, Jorge Ricardo-da-Silva, Ricardo Chagas, Luísa Maria Ferreira

**Affiliations:** 1 LEAF—Linking Landscape, Environment, Agriculture and Food Research Center, Instituto Superior de Agronomia, Universidade de Lisboa, Tapada da Ajuda, 1349-017 Lisboa, Portugal; f.saracino19@icloud.com (F.S.); rbferreira@isa.ulisboa.pt (R.B.F.); jricardosil@isa.ulisboa.pt (J.R.-d.-S.); 2 CENSE—Center for Environmental and Sustainability Research, NOVA School of Science and Technology, NOVA University of Lisbon, 2829-516 Caparica, Portugal; j.brinco@campus.fct.unl.pt; 3 LAQV-REQUIMTE, Departamento de Química, NOVA School of Science and Technology, 2829-516 Caparica, Portugal; dx.gago@campus.fct.unl.pt (D.G.); mdr@fct.unl.pt (M.G.d.S.); ricardo.chagas@food4sustainability.org (R.C.); 4 Food4Sustainability—Associação Para a Inovação no Alimento Sustentável, Centro Empresarial de Idanha-a-Nova, Zona Industrial, 6060-182 Idanha-a-Nova, Portugal

**Keywords:** white wine, DCMC, bentonite, protein stability, wine protein, wine aromatic fraction

## Abstract

Protein haze in white wine is one of the most common non-microbial defects of commercial wines, with bentonite being the main solution utilized by the winemaking industry to tackle this problem. Bentonite presents some serious disadvantages, and several alternatives have been proposed. Here, an alternative based on a new cellulose derivative (dicarboxymethyl cellulose, DCMC) is proposed. To determine the efficiency of DCMC as a bentonite alternative, three monovarietal wines were characterized, and their protein instability and content determined by a heat stability test (HST) and the Bradford method, respectively. The wines were treated with DCMC to achieve stable wines, as shown by the HST, and the efficacy of the treatments was assessed by determining, before and after treatment, the wine content in protein, phenolic compounds, sodium, calcium, and volatile organic compounds (VOCs) as well as the wine pH. DCMC applied at dosages such as those commonly employed for bentonite was able to reduce the protein content in all tested wines and to stabilize all but the Moscatel de Setúbal varietal wine. In general, DCMC was shown to induce lower changes in the wine pH and phenolic content than bentonite, reducing the wine calcium content. Regarding which VOCs are concerned, DCMC produced a general impact similar to that of bentonite, with differences depending on wine variety. The results obtained suggest that DCMC can be a sustainable alternative to bentonite in protein white wine stabilization.

## 1. Introduction

Proteins are present in wines in relatively low levels. These essentially comprise plant defense proteins and their concentration in wines is not only dependent on the grape composition (including protein content), but also on the grape variety, maturation conditions [1], and winemaking process as well as on the environmental conditions prevailing during vegetative growth [1,2]. Protein haze in white wines, where limpidity is an essential sensory quality parameter, is one of the most common non-microbial defects of commercial wines [3]. These proteins can be responsible for wine colloidal instability, forming amorphous sediments or flocculates, and producing a suspended and undesirable haze, before or after bottling, which can cause serious economic losses to the wine producers [4].

The best commercially available solution for protein haze in the winemaking industry is bentonite, a montmorillonite clay, negatively charged at the wine pH, which removes proteins based on charge interactions and physical adsorption [5]. While bentonite is a natural clay material, it must be mined from special deposits, which limits the sustainability of the application. The use of bentonite presents additional disadvantages comprising: (i) the handling of dust before the application due to the health hazards associated with it; (ii) the disposal of used bentonite; (iii) the direct adsorption of aroma compounds on the bentonite clay, which severely affects the sensory profile of the wine; and (iv) the loss of wine in the form of bentonite lees [6]. Therefore, there is a significant focus on developing alternative economical practices to replace bentonite to stabilize wines. Some of these state-of-the-art practices include the use of magnetic nanoparticles [7], free [8] and immobilized proteases [9], chitosan [10] or chitin-rich yeasts [6], zirconia [11] or low-swelling adsorbing clays [12], together with techniques such as ultrafiltration [13] or flash pasteurization [14].

The loss of aromas resulting from bentonite fining, widely used since 1950, has been reported several times by the professionals [15], but the induced loss is poorly quantified. Even if some reports suggest that bentonite fining can have a lower impact on the aroma quality when used before fermentation, the direct adsorption of the aroma molecules is responsible for the aroma losses after fining [16]. A recent work on two Portuguese white monovarietal wines also reported that the volatile organic compound (VOC) profile is highly impacted by using or not using bentonite fining, an observation that is variety-dependent [17].

Dicarboxymethyl cellulose (DCMC) is a recently described cellulose-derived polymer [18], which is negatively charged at the wine pH and presents the capacity to adsorb positively charged substances [19] and wine proteins [20]. It was therefore of interest to apply DCMC to wines, especially to those prepared from cultivars that are generally known to produce high protein instability wines. The aim of this work was to compare the efficacy of DCMC with that of bentonite in decreasing the hazing potential of white wines and impact on the overall aroma. For this purpose, the presence of proteins in wines, together with other physico-chemical proprieties were recorded, before and after each treatment, in relation to their aroma profiles.

## 2. Results and Discussion

### 2.1. Wine Characterization

Differences in wine turbidity (before and after the heat treatment) have been shown to correlate directly to wine protein instability, with the value of 0.02 of the pass-fail point in protein stability tests suggested by several authors [21,22]. Moscatel de Setúbal, Viosinho, and Encruzado varietal wines were submitted to the heat stability test (HST) resulting in a highly unstable (Moscatel de Setúbal), a moderately unstable (Viosinho), and another one that coincided with the stability threshold wine (Encruzado) (Table 1). Protein content of the wines was determined by the Bradford method [23].

The three wines under study were subjected to routine analyses to obtain an identity card for each of them. The results of the routine analyses, which were within the expected ranges, are presented in the Supplementary Materials (Table S1).

Knowing that protein concentration alone is not enough to explain the protein instability of a wine [24], it has been shown that, in general, protein concentration is more influential on the amount of haze produced than protein composition [25], even if this supposition is questionable if taking in account that the identification of SO_2_ as the modulator factor makes protein haze formation dependent on the presence of sulfydryl-rich wine proteins such as the thaumatin-like proteins [21,24]. To quantify the protein in the wine samples before and after the stabilizing treatments, the Bradford protein assay [23] was used to estimate the total proteins present, as shown in Table 1 for the wines before any stabilizing treatment. From this analysis, the Moscatel de Setúbal varietal wine has a higher protein content, while Viosinho and Encruzado showed lower contents, all in a concentration range described as common for untreated wines [4].

### 2.2. Effects of Fining Treatments on Wine Stability and Protein Content

**Figure 1:**
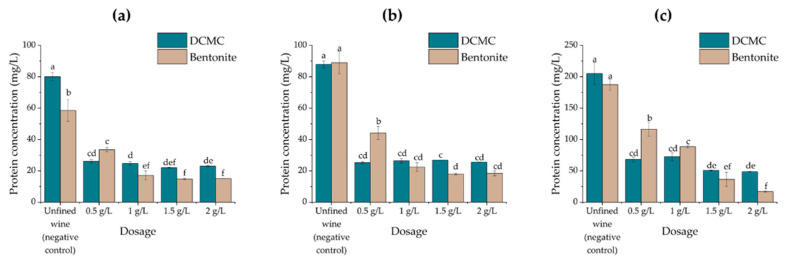
Protein concentration (mg/L; quantified by the Bradford method) in wine samples treated with increasing amounts of bentonite or DCMC. Different letters represent statistically significant differences among different homogeneous subsets for *p* = 0.05. (**a**) Encruzado, (**b**) Viosinho, and (**c**) Moscatel de Setúbal monovarietal wines.

**Figure 2:**
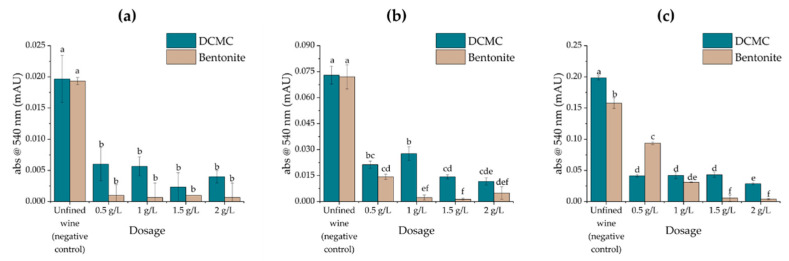
Turbidity (following a HST) of wines treated with increasing amounts of bentonite or DCMC. Different letters represent statistically significative differences among different homogeneous subsets for *p* = 0.05. (**a**) Encruzado, (**b**) Viosinho, and (**c**) Moscatel de Setúbal monovarietal wines.

The three unfined white wines under analysis were treated with dicarboxymethyl cellulose (DCMC) or bentonite at four different dosage rates (0.5, 1, 1.5, and 2 g/L) commonly employed on commercial wines, even if the lower effective dose was the objective [26]. The wines were treated for 48 h and the remaining soluble protein was quantified using a Macro-Bradford assay. The haze forming potential of the wines was subsequently assessed by a HST (Figure 1 and Figure 2).

As expected, the use of increasing concentrations of both DCMC and bentonite in wines promotes the reduction in protein concentration, with DCMC more effective than bentonite at lower doses of fining agent used (0.5 g/L), but less effective at all the higher doses used (1.0, 1.5, and 2.0 g/L) on the three wines, except in the case of Moscatel de Setúbal at 1.0 g/L of fining agent, where the varietal wine contained a very high protein concentration (near 200 mg/L) before the treatments. Since both agents presented negative electrical charge at the wine pH, the results obtained may vary based on the different surfaces exposed by DCMC and bentonite, since the latter has the ability to accommodate harder proteins, usually less prone to absorb, but also more likely to promote haze formation [26].

**Figure 3:**
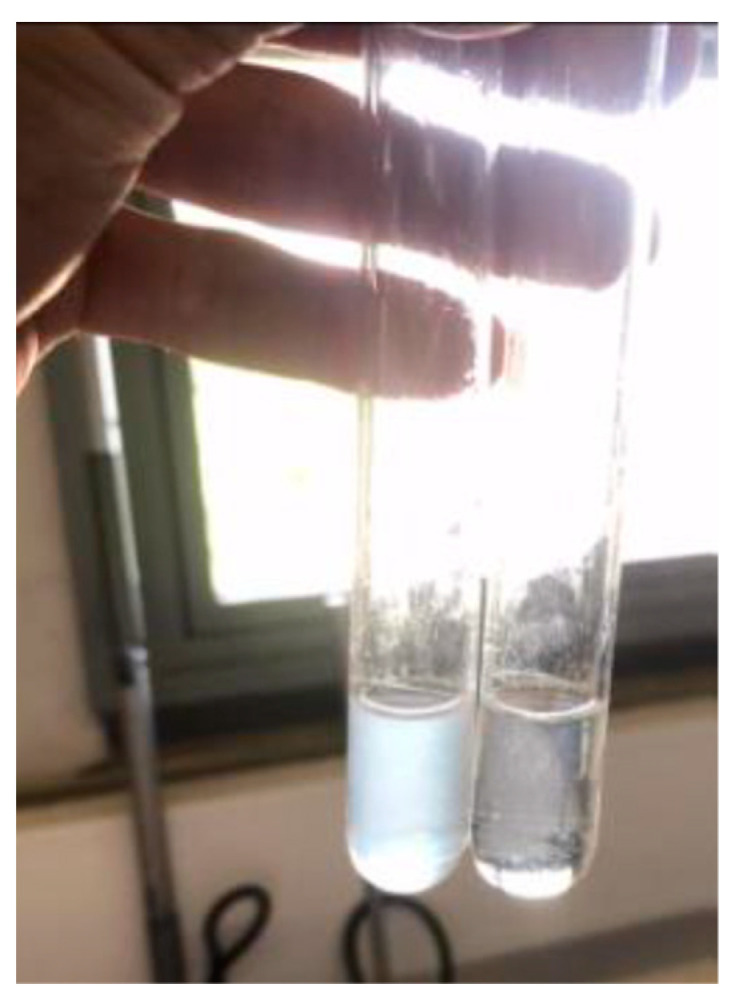
Untreated (**left**) and treated (**right**) with 0.5 g/L of DCMC samples of Moscatel de Setúbal wine following a HST.

Concerning the HST results, while the samples from the varietal wines Encruzado and Viosinho were stabilized by bentonite and DCMC at all tested dosages, the wine Moscatel de Setúbal required doses higher than 1.5 g/L of bentonite or, even with a very low turbidity formation after the HST (Figure 3), it never achieved full stability (the difference in A_540_ values before and after HST were greater than 0.02) after DCMC treatment.

### 2.3. Effect of Wine Fining Treatments on Wine Chemical Composition

**Figure 4:**
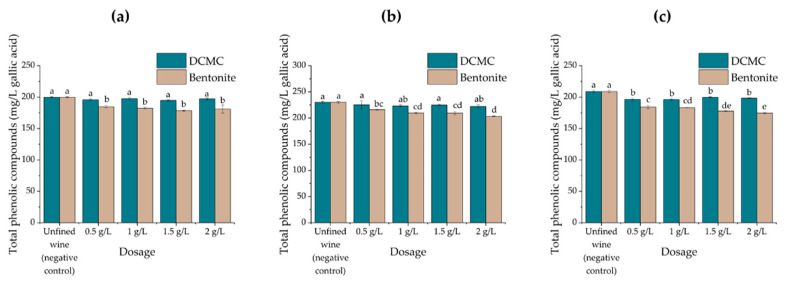
Changes in the amount of total wine phenolics detected by the absorbance at 280 nm for increasing amounts of bentonite or DCMC applied to samples of the wines under study. Different letters represent statistically significant differences among different homogeneous subsets for *p* = 0.05. (**a**) Encruzado, (**b**) Viosinho, and (**c**) Moscatel de Setúbal monovarietal wines.

As described before [27], bentonite fining in wines always resulted in a significant decrease in total phenolic concentration. In contrast, the DCMC treatment had little influence on the wine phenolic content and for any dose tested, it only removed a smaller fraction of the phenolic compounds (Figure 4).

**Figure 5:**
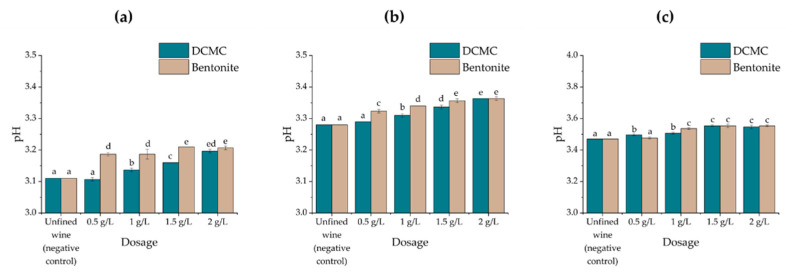
Changes in the wine pH after treatment with increasing amounts of bentonite or DCMC. Different letters represent statistically significative differences among different homogeneous subsets for *p* = 0.05. (**a**) Encruzado, (**b**) Viosinho, and (**c**) Moscatel de Setúbal monovarietal wines.

For all wine samples treated as well as in the controls, the pH was measured before and after treatment to evaluate the potential effect exerted by bentonite or DCMC. In all trials, the maximum increase in pH, after each treatment, was 0.1 pH units. In the Encruzado and Viosinho sample wines, DCMC had a significantly smaller impact on the wine pH than bentonite, but exerted a mixed pattern in the highly unstable wine Moscatel de Setúbal (Figure 5).

Protein removal by cationic exchange typically results in increasing the wine content in inorganic cations. In bentonite, the negative net charge of the natural clay is partially balanced by exchangeable cations, mainly Ca^2+^, K^+^, and Mg^2+^, and the commercially available products are usually modified through an activation process, which enriches the calcium-dominant clay with sodium [28]. Calcium can contribute to tartaric instability since calcium tartrate is 10 times less soluble than potassium bitartrate [15].

**Figure 6:**
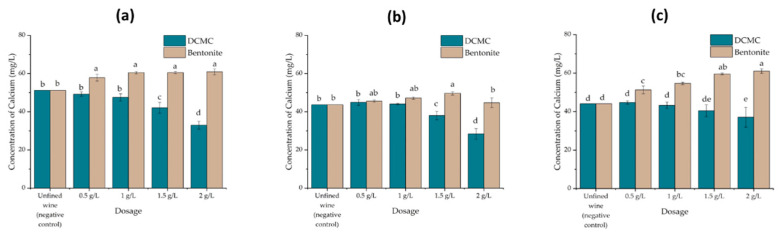
Changes in calcium wine content as determined by ICP-AES on treated and untreated wine samples with increasing amounts of bentonite or DCMC. Different letters represent statistically significant differences among different homogeneous subsets for *p* = 0.05. (**a**) Encruzado, (**b**) Viosinho, and (**c**) Moscatel de Setúbal monovarietal wines.

For all three varietal wines under analysis, DCMC produced significant differences in the decrease in wine calcium content when compared to bentonite, producing significant differences when compared to untreated and treated with bentonite wines (Figure 6). In contrast, wines treated with bentonite always showed an increase in calcium content, in contrast to DCMC-treated wines in which wine calcium decreased with increasing DCMC applied. The increase in wine sodium content by treatment with DCMC was not determined once DCMC is an organic compound with a low content in sodium (0.62%). The maximum amount of sodium that this DCMC can transfer to wine in a dosage of 2 g/L of wine is 12.4 mg of sodium, which is irrelevant for the wine quality.

DCMC has a high capacity to remove positively charged molecules from solution, therefore, it can carry out a cation exchange involving the removal of proteins and is also able to remove calcium. This decrease may result in more stable wines in terms of tartaric stabilization.

### 2.4. Effect of Wine Fining Treatments in the Profile of Volatile Organic Compounds

During fermentation, yeast metabolizes nutrients in order to support growth and thus produce biomass. This complex metabolic activity generates a variety of volatile organic compounds (VOCs), which are released during the fermentation process. For the analysis of VOCs, three replicate samples from each monovarietal wine were treated with several concentrations of bentonite, DCMC, and a control (i.e., non-treated) sample. A commercial hydrocarbon mixture (C8–C20) was used to calculate the linear retention indices (LRI) and compared with the LRI values available in the literature [17,29,30,31]. A total of 71 compounds was detected, with a signal-to-noise ratio above 10 when ion extraction of the most abundant ionic form of each compound was performed. Of those, 61 were tentatively identified by matching mass spectra with the spectra of reference compounds in the NIST 17 mass spectra database and LRI data from the literature (Table 2). These compounds represent over 90% of the total ion chromatogram (TIC) area.

**Figure 7:**
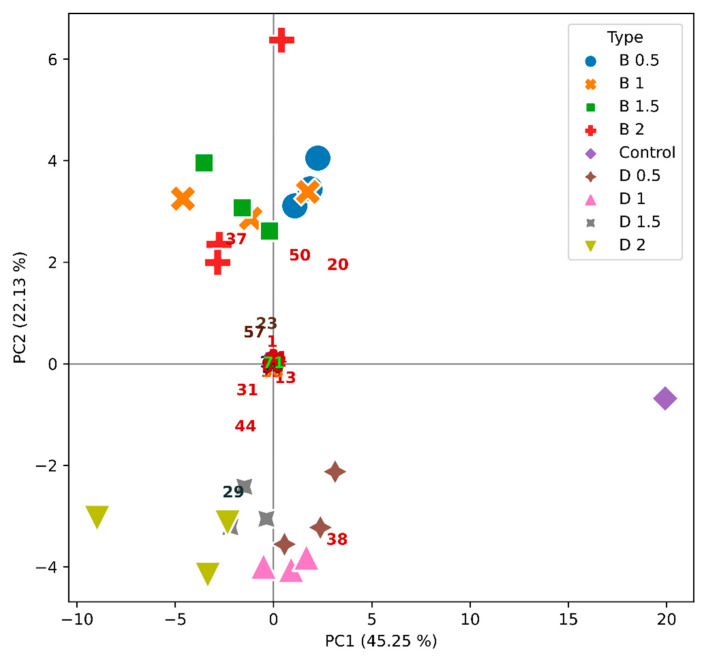
PCA illustrating the simultaneous projection of the wine samples (objects)—by a mark, and VOCs (variables) loadings—by a number, for the Encruzado varietal wine. The different concentrations of bentonite (B) or DCMC (D) in g/L are indicated.

**Figure 8:**
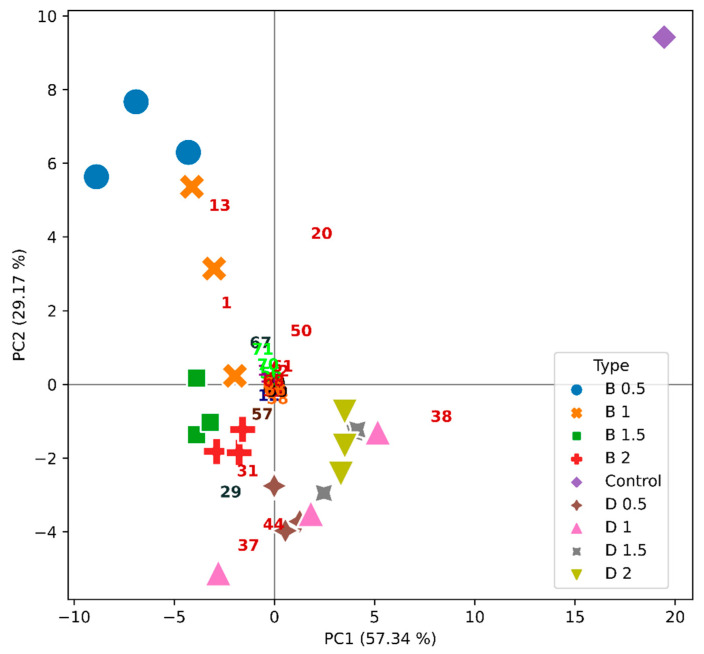
PCA illustrating the simultaneous projection of the wine samples (objects)—by a mark, and VOC (variables) loadings—by a number, for the Viosinho varietal wine. The different concentrations of bentonite (B) or DCMC (D) in g/L are indicated.

**Figure 9:**
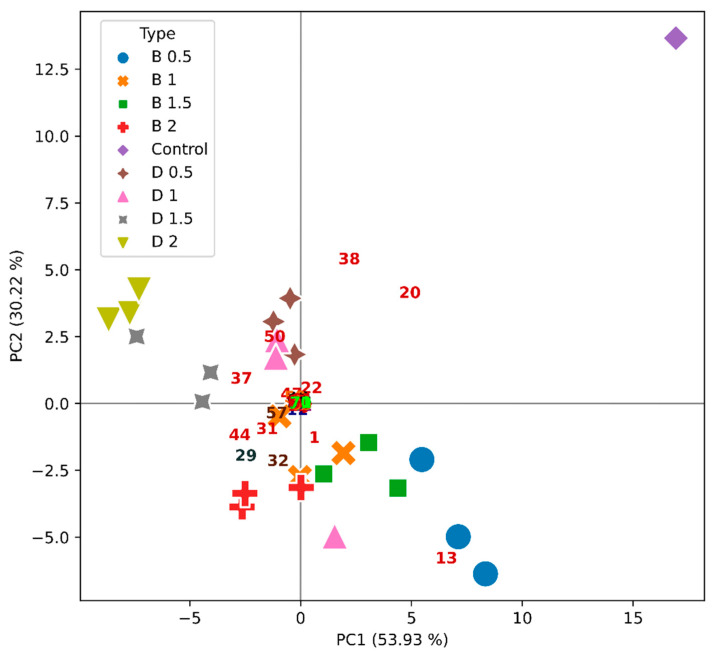
PCA illustrating the simultaneous projection of the wine samples (objects)—by a mark, and VOC (variables) loadings—by a number, for the Moscatel de Setúbal varietal wine. The different concentrations of bentonite (B) or DCMC (D) in g/L are indicated.

Esters were the most abundant group, with 22 compounds. Alcohols and carbonyl compounds were also detected, along with terpenoids and alkanes, as reported elsewhere for Portuguese wines varieties [17,30,31,54,55]. Considering the extensive chromatographic data generated, a multivariate technique of data analysis was used. Indeed, principal component analysis (PCA) is considered an appropriate statistical method, which allows us to reveal basal data structures when hidden patterns are easier to perceive [17,30,31,56]. In Figure 7, Figure 8 and Figure 9, one can observe that all the monovarietal wines studied could be identified through their VOC profile when polymer or bentonite was used.

For Encruzado (Figure 7), the first and second PCs explained more than 67% of the system variance. The samples treated with bentonite were clearly separated from the samples treated with the polymer through PC2, namely by peaks **1** (ethyl acetate), **20** (ethyl hexanoate), **23** (limonene), **37** (diethyl succinate), **50** (ethyl decanoate), and **57** (2,4-di-tert-butylphenol), whereas the DCMC-treated samples were characterized by peaks **29** (4-methyl benzaldehyde) and **44** (2-phenethyl acetate). The control was separated through peaks **20** (ethyl hexanoate) and **38** (ethyl octanoate) from all other Encruzado samples along PC1.

For Viosinho (Figure 8), the first and second PCs explained more than 86% of the system variance. The samples treated with bentonite were separated from the samples treated with DCMC through the second PC. In fact, peaks **1** (ethyl acetate), **13** (isoamyl acetate), **20** (ethyl hexanoate), **50** (ethyl decanoate), **57** (2,4-di-tert-butylphenol), **61** (ethyl dodecanoate), **67** (heptadecane), **70** (nonadecane), and **71** (eicosane) separate this monovarietal wine treated with bentonite from the DCMC-treated wine samples, which were characterized by peaks **29** (4-methyl benzaldehyde), **31** (2-butoxyethyl acetate), **37** (diethyl succinate), and **44** (2-phenethyl acetate). The control was separated through peak **38** (ethyl octanoate) from all other Viosinho samples along PC1.

For Moscatel de Setúbal (Figure 9), PC1 and PC2 were responsible for more than 84% of the system variance. Samples treated with DCMC were characterized by peaks **22** (hexyl acetate), **31** (2-butoxyethyl acetate), **37** (diethyl succinate), **44** (2-phenethyl acetate), **50** (ethyl decanoate), and **57** (2,4-di-tert-butylphenol) along PC2, whereas samples treated with bentonite were characterized by peaks **1** (ethyl acetate), **13** (isoamyl acetate), **29** (4-methyl benzaldehyde), and **32** (ethyl 3-(methylthio)-propanoate), also mainly along the PC2. Once again, the control was separated through peaks **20** (ethyl hexanoate) and **38** (ethyl octanoate) from all other Moscatel de Setúbal-treated samples along PC2.

**Figure 10:**
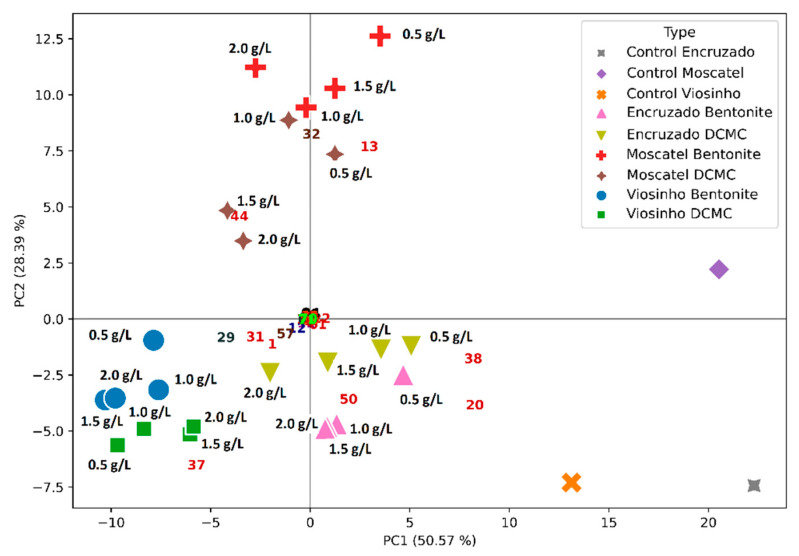
PCA illustrating the simultaneous projection of the wine samples (objects)—by a mark, and VOC (variables) loadings—by a number, for all wine samples under study. The different concentrations of bentonite or DCMC in g/L are indicated next to each object mark, which represents the mean value of the three replicates.

The above PCA results for each monovarietal wine seem to indicate that significant differences are possible to identify between bentonite- and DCMC-treated samples and the respective controls, mainly by peaks **20** (ethyl hexanoate) and **38** (ethyl octanoate). Actually, both these compounds decreased in quantity when the two fining agents (i.e., bentonite or DCMCO) were used, as also observed by Santos el al. [17] for the Síria and Arinto wines when the addition of bentonite was under study. It has been proposed that removal of these long chain esters by negatively charged bentonite results from a synergetic effect of the adsorption of the esters to the clay and proteins. The same should happen in the presence of DCMC. Nevertheless, it is not clear from the individual PCAs (Figure 7, Figure 8 and Figure 9) whether the VOC profiles exert a differential effect in the aroma of the wines when bentonite or DMMC are used. Thus, in Figure 10, all monovarietal wines and controls are represented (only mean values were considered for the sake of clarity). PC1 and PC2 explained almost 79% of the system variance. Controls of the three varietal wines were completely separated from the varietal wines treated with DCMC and bentonite through the first PC, by peaks **20** (ethyl hexanoate) and **38** (ethyl octanoate). The first PC also separated Viosinho from Encruzado, mainly by peaks **29** (4-methyl benzaldehyde) and **37** (diethyl succinate), which characterized Viosinho, and peak **50** (ethyl decanoate) characterizing Encruzado. Moscatel de Setúbal varietal wine was separated from the other varietal wines through the second PC, by peaks **13** (isoamyl acetate), **32** (ethyl 3-(methylthio)-propanoate), and **44** (2-phenethyl acetate) and within the same varietal wine between the bentonite and DCMC treatments. The second PC also separated Viosinho from Encruzado, regardless of using bentonite or polymer. It may appear that the separation obtained was more impacted by the varietal wine than by the bentonite or DCMC treatments. Within each varietal wine, the use of bentonite or polymer does not indicate a significant change in the VOC profiles obtained. It is worth noting that for each varietal wine, the VOC profiles and treatments were concentration-dependent, but less dependent on the fining agent used (i.e., bentonite or DCMC). Indeed, in Figure 10, this is clear for Viosinho and Encruzado, though less evident for Moscatel de Setúbal.

The capacity to promote protein removal, the phenolic content and pH preservation, the slight calcium depletion and predictable changes in the wine aromatic fraction allowed us to propose DCMC as a sustainable alternative to bentonite fining in white wine protein stabilization.

## 3. Materials and Methods

### 3.1. Reagents and Materials

All commercial chemical reagents and solvents were purchased from Sigma-Aldrich (Darmstadt, Germany). Bradford reagent (Quick Start Bradford 1× Dye Reagent) was purchased from Bio-Rad (Hercules, CA, USA). Bentonite was used as sodium bentonite (Enartis).

The dicarboxymethyl cellulose (DCMC) used was prepared as described elsewhere [19] and presented 0.27 mmol of carboxylate groups per gram of polymer.

### 3.2. Wines

Monovarietal wines were prepared from three different white grape varieties at the winery of Instituto Superior de Agronomia, University of Lisbon, Lisbon, Portugal using the classic white winemaking technology. Specifically, the varieties were Encruzado, Viosinho, and Moscatel of Setúbal, all *Vitis vinifera* L., from the 2018 harvest at the Instituto Superior de Agronomia, at technical grape maturity. The wine samples were always stored in a dark room, in bottles filled up with nitrogen.

### 3.3. Heat Stability Test (HST)

Wine samples were heated at 80 °C for 2 h in a thermomixer and subsequently cooled in ice for 2 h. After equilibration at ambient temperature, the increase in turbidity was detected spectrophotometrically at 540 nm. Differences in wine turbidity (before and after the heat treatment) have been shown to correlate directly to wine protein instability. The value of 0.02 AU was used as the pass-fail point in the protein stability tests [21,22]. All measurements were performed in triplicate.

### 3.4. Analytical Enological Parameters

Several analytical enological parameters were determined in the wine samples following the International Organization of Wine and Vine (2016) guidelines. In more detail, reduced sugar content (g/L), alcohol content (% *v/v*), titratable acidity (expressed as g tartaric acid/L), volatile acidity (expressed as g acetic acid/L), free and total SO_2_ content (mg/L), density (g/mL), dry matter (g/L), color intensity (AU), total phenolics (expressed in mg of gallic acid/L) differentiated in flavonoids and non-flavonoids [57,58], Cl (mg NaCl/L), Cu (mg/L), Fe (mg/L), Ca (mg/L), Mg (mg/L), Na (mg/L), K (mg/L), sulfates (g of potassium sulfate/L), tartaric stability (% of conductivity dropping), and pH were measured. All measurements were performed in duplicate or triplicate. All parameters measured are presented in the Supplementary Materials.

Calcium was also analyzed in treated and untreated wine samples by inductively coupled plasma atomic emission spectroscopy (ICP-AES) using a Horiba Jobin-Yvon Ultima model equipped with a 40.68 MHz RF generator, a Czerny–Turner monochromator with 1.00 m (sequential), and an autosampler AS500.

### 3.5. Protein Quantification

Protein quantification in the wine samples was performed by the Bradford method, with minor modifications. A calibration curve was prepared with bovine serum albumin (BSA) as the standard, using the following concentrations: 2.5 μg/mL, 5 μg/mL, 7.5 μg/mL, 10 μg/mL, 15 μg/mL, and 20 μg/mL.

Protein samples (400 μL aliquots) dissolved in the various matrices were mixed with an equal volume of deionized water, to which 200 μL of the Bio-Rad Protein assay reagent was added. The absorbance at 595 nm was taken after holding the samples at room temperature for 10 min. All measurements were performed in triplicate [59].

### 3.6. Fining Experiments

Synthesized DCMC and commercial bentonite were used as fining agents in the selected wine samples. The fining experiments involved the addition of standard concentrations of DCMC, followed by comparison with the wines treated with the same dosages of bentonite. The trials were performed at a laboratory scale using 20 mL aliquots of wine. The unfined wines were used as negative controls. DCMC and bentonite were added to wines previously clarified by centrifugation at 10,000× *g* for 15 min and then incubated for 48 h at 25 °C under agitation. The samples were then centrifuged at 10,000× *g* for 15 min and filtered to remove residual amounts of the fining agents. All trials were performed in triplicate.

### 3.7. Determination of Wine Volatile Organic Compounds

A carboxen/divinylbenzene/polydimethylsiloxane fiber (DVB/Carb/PDMS, 1 cm, 50/30 μm film thickness (df)) supplied by Supelco (Bellefonte, PA, USA) was used for HS-SPME extractions, following the methodology previously described [30]. Before use, the fiber was conditioned, following the manufacturer’s recommendations. Fiber blanks were run periodically to ensure the absence of contaminants and carryover. Five mL of wine was transferred to a 20 mL vial containing 0.6 g NaCl and placed in a LECO L-PAL3 autosampler. The vial was incubated at 30 °C for 3 min and then extracted for 30 min at the same temperature. The fiber was exposed to the GC inlet for 3 min. Regular blank (lab air) samples were made to confirm the absence of sample carryover. GC-TOFMS analyses were performed with an Agilent 7890B (Palo Alto, CA, USA) gas chromatograph equipped with a split/splitless injector. An Agilent HP-5MS UI fused silica capillary column (30 m × 0.25 mm i.d., 0.25 µm df) was used for all separations. The injector was set at 250 °C in splitless mode for 60 s, then purged with 20 mL/min. Helium was used at a constant flow rate of 1 mL/min. The oven program was as follows: 40 °C for 4 min, then 4 °C/min until 220 °C, then 20 °C/min until 250 °C, for a total run time of 50.5 min, plus cooling. Detection was performed with a LECO Pegasus BT Time-of-Flight mass spectrometer (Saint Joseph, Michigan, USA). The transfer line was at 250 °C. The MS was operated with the ion source at 250 °C, electron ionization at 70 eV, acquisition from *m/z* 40 to 350 Da, 10 spectra per s, and an acquisition delay of 2 min. Data acquisition, system control, and spectra deconvolution were performed using LECO ChromaTOF version 5.40. NIST MS Search Program Version 2.3 g was used for spectra matching. Linear retention index (LRIs) values for sample peaks were calculated by analyzing the commercial alkane standard solution C8–C20 using the aforementioned chromatographic conditions.

### 3.8. Statistical Analyses

Results were averages of two or three measurements, obtained from three replicate treatments. Standard deviations were also calculated and significant differences among samples were assessed at *p* < 0.05, by one-way ANOVA and the Tukey honest significant difference test (HSD test). The variances were homogeneous for the Levene and Brown–Forsythe tests. For the aroma analyses, the data were subjected to principal component analysis (PCA). To study the correlation among the different instabilities of wines and their protein concentrations, R-project 3.4.3 was used.

For PCA analysis, the relative percent area of each peak was used, which assumes the sum of all peaks in a chromatogram to be 100%. Integration was performed on the *m*/*z* with the highest signal-to-noise ratio for each compound. Calculation and graphing were performed in Python (Version 3.9.5) using the following libraries: Scikit-learn (Version 0.24), namely the PCA class in the decomposition module (sklearn.decomposition.PCA); Pandas (Version 1.2.5); Seaborn (Version 0.11.1); and Matplotlib (Version 3.4.2).

## Figures and Tables

**Table 1:** Results of the protein concentration and protein stability tests (HST).

Wine	Protein Concentration (mg/L)	HST	Stability ^1^
Encruzado	80.1 ± 8	0.019 ± 0.002	stable
Viosinho	87.9 ± 6	0.073 ± 0.008	unstable
Moscatel de Setúbal	218.7 ± 10	0.190 ± 0.01	unstable

^1^ A wine is considered unstable if the difference in wine absorbance at 540 nm (before and after the heat treatment) is higher than 0.02 AU. Values shown are mean ± SD (*n* = 3).

**Table 2:** VOCs tentatively identified in the samples under analysis by GC/TOFMS.

Peak #	Chemical Family	Compound Name	LRI _Calc_^a^	LRI _Lit_^a^*	Δ (LRI _Calc_-LRI _Lit_)
6	Alcohol	3-Methyl-1-pentan-1-ol	851	843	8
9	Alcohol	3-Hexen-1-ol (isomer)	860	855	5
12	Alcohol	Hexan-1-ol	872	870	2
24	Alcohol	2-Ethylhexanol	1030	1029	1
25	Alcohol	Benzyl alcohol	1033	1033	0
28	Alcohol	Octan-1-ol	1071	1078	−7
29	Aldehyde	4-Methyl benzaldehyde	1078	1079	−1
39	Aldehyde	Decanal	1203	1205	−2
5	Alkanes	2,4-Dimethyl-hept-1-ene	845	842	3
33	Alkanes	Cosmene	1129	1134	−5
48	Alkanes	Tridecane	1296	1300	−4
56	Alkanes	Pentadecane	1498	1500	−2
67	Alkanes	Heptadecane	1693	1700	−7
70	Alkanes	Nonadecane	1895	1900	−5
71	Alkanes	Eicosane	1995	2000	−5
10	Aromatic	Ethylbenzene	862	864	−2
11	Aromatic	Xylene	869	866	3
19	Aromatic	1,3,5-Trimethyl-benzene	990	995	−5
55	Aromatic	3-Ethyl-3-phenyl-pent-1-ene	1477		NC
1	Ester	Ethyl acetate	NC	610	NC
2	Ester	Ethyl isobutyrate	NC	756	NC
3	Ester	Ethyl butanoate	814	806	8
4	Ester	Ethyl lactate	824	815	9
7	Ester	Ethyl 2-methylbutanoate	856	856	0
8	Ester	Ethyl 3-methylbutanoate	859	867	−8
13	Ester	Isoamyl acetate	880	878	2
15	Ester	1-Ethoxypropan-2-yl acetate	937	965	−28
20	Ester	Ethyl hexanoate	1000	1001	−1
22	Ester	Hexyl acetate	1014	1015	−1
31	Ester	2-Butoxyethyl acetate	1093	1090	3
37	Ester	Diethyl succinate	1181	1182	−1
38	Ester	Ethyl octanoate	1196	1199	−3
43	Ester	Ethyl phenylacetate	1242	1244	−2
44	Ester	2-Phenethyl acetate	1253	1257	−4
47	Ester	Ethyl nonanoate	1293	1294	−1
50	Ester	Ethyl decanoate	1394	1397	−3
52	Ester	Ethyl-3-methylbutyl butanedioate	1428	1433	−5
61	Ester	Ethyl dodecanoate	1589	1597	−8
62	Ester	1-*O*-(2-Methylpropyl) 4-*O*-propan-2-yl-2,2-dimethyl-3-propan-2-yl-butanedioate	1592	1581	11
68	Ester	Ethyl tetradecanoate	1789	1793	−4
14	Ketone	6-Methyl-2-heptanone	928	932	−4
27	Ketone	2-Nonanone	1055	1091	−36
54	Ketone	Geranyl acetone	1451	1452	−1
17	Miscellaneous	3-(Methylthio)-1-propanol (methionol)	977	981	−4
18	Miscellaneous	3(2H)-Thiophenone, dihydro-2-methyl-	984	996	−12
32	Miscellaneous	ethyl 3-(methylthio)-propanoate	1100	1098	2
45	Miscellaneous	Vitispirane	1275	1281	−6
49	Miscellaneous	Isobutyl-2,2,4-trimethyl-3-hydroxy-pentanoate	1348		NC
57	Miscellaneous	2,4-Di-tert-butylphenol	1511	1513	−2
59	Miscellaneous	Dehydro-ar-himachalene	1537	1514	23
23	Terpenoid	Limonene	1026	1028	−2
26	Terpenoid	β-Ocimene	1038	1049	−11
30	Terpenoid	Terpinolene	1086	1088	−2
34	Terpenoid	Nerol oxide	1153	1153	0
36	Terpenoid	Linalool	1171	1099	72
40	Terpenoid	Nerol	1226	1228	−2
46	Terpenoid	Geraniol	1285	1267	18
60	Terpenoid	α-Calacorene	1540	1542	−2
63	Terpenoid	α-Corocalene	1617	1623	−6
16	Unknown	Unknown	971		
35	Unknown	Unknown	1167		
41	Unknown	Unknown	1231		
42	Unknown	Unknown	1236		
51	Unknown	Unknown	1415		
58	Unknown	Unknown	1519		
64	Unknown	Unknown	1639		
65	Unknown	Unknown	1658		
66	Unknown	Unknown	1663		

^a^ LRI_calc_—retention indices calculated from C8—C20 *n*-linear alkanes, LRI_Lit_—linear retention indices reported in the literature for DB5 capillary column [17,29,30,31,32,33,34,35,36,37,38,39,40,41,42,43,44,45,46,47,48,49,50,51,52,53], NC—not calculated.

## Data Availability

Not applicable.

## Supplementary Materials

The following material is available online, Table S1: Routine analyses of the three wines from 2018 under study.

## Sample Availability

Samples of DCMC are available from the authors.
