# Supplementary material for: DCMC as a Promising Alternative to Bentonite in White Wine Stabilization. Impact on Protein Stability and Wine Aromatic Fraction
Source: Molecules. 2021 Oct 14;26(20):6188. doi: 10.3390/molecules26206188 (PMC8539232; doi:10.3390/molecules26206188)
Supplement: Supplementary file 1 [file molecules-26-06188-s001.zip › molecules-1401048-supplementary.pdf]

Table S1. -Routine analyses of the three 2018 studied wines; values are mean ± SD (n = 3).

Wine	Free SO_2 (mg/L)	Total SO_2 (mg/L)	pH	Total acidity (g/L of tartaric acid)	Volatile acidity (g/L of acetic acid)	Alcohol (% v/v)	Reducing substances (g/L)
Encruzado	25 ± 0	90 ± 0	3.12 ± 0	8.4 ± 0.11	0.28 ± 0.03	13.5 ± 0.01	0.1 ± 0
Viosinho	28 ± 0	82 ± 0	3.28 ± 0	7.4 ± 0.06	0.4 ± 0.01	16 ± 0.02	1.2 ± 0.01
Moscatel de Setúbal	21 ± 0	80 ± 0	3.47 ± 0	6 ± 0.11	0.32 ± 0.01	14.1 ± 0.0	0.43 ± 0.02

Wine	Dry matter (g/L)	Colour (AU)	Total phenols (mg/L of gallic acid)	Non-flavonoids (mg/L of gallic acid)	Flavonoids (mg/L of gallic acid)	Chloride (mg NaCl/L)	Sulphates (g/L of potassium sulphate)
Encruzado	22.2 ± 0.18	0.067 ± 0	192.6 ± 1.4	67.8 ± 1.4	124.8 ± 1	8.12 ± 0.1	0.2 ± 0.03
Viosinho	23.6 ± 0.35	0.066 ± 0	227.5 ± 3.5	77.5 ± 1.1	149.6 ± 1.7	12.72 ± 0.18	0.2 ± 0.01
Moscatel de Setúbal	21.4 ± 0.1	0.048 ± 0	189.6 ± 1.8	68.0 ± 1.7	121.6 ± 2.3	10.97 ± 0.13	0.1 ± 0.01

Wine	Tartaric stability (%)	Cu (mg/L)	Fe (mg/L)	Ca (mg/L)	Mg (mg/L)	Na (mg/L)	K(mg/L)
Encruzado	10.7 ± 1	<0.01 ± 0	0.75 ± 0.01	55.59 ± 1	68.42 ± 0.9	6.76 ± 0.17	756.57 ± 3
Viosinho	3.6 ± 0.8	<0.01 ± 0	0.81 ± 0.01	44.13 ± 0.5	87.49 ± 0.8	9.85 ± 0.25	525.04 ± 1.5
Moscatel de Setúbal	7.8 ± 0.07	0.062 ± 0	0.60 ± 0.01	42.78 ± 0.45	74.50 ± 0.5 7	7.63 ± 0.25	808.60 ± 2.5
